# Supplementary material for: Spatial patterning of fibroblast TGFβ signaling underlies treatment resistance in rheumatoid arthritis
Source: bioRxiv. 2025 Mar 17:2025.03.14.642821. Preprint. [Version 1] doi: 10.1101/2025.03.14.642821 (PMC11956964; doi:10.1101/2025.03.14.642821)
Supplement: 1 [file NIHPP2025.03.14.642821V1-supplement-1.pdf]

bioRxiv preprint doi: https://doi.org/10.1101/2025.03.14.642821; this version posted March 17, 2025. The copyright holder for this preprint (which was not certified by peer review) is the author/funder, who has granted bioRxiv a license to display the preprint in perpetuity. It is made available under aCC-BY 4.0 International license.

	Total	remission	non-remission	borderline
	(N=17)	(N=10)	(N=6)	(N=1)
**Age (y)**				
Mean (SD)	47.4 (17.4)	50.3 (22.1)	44.8 (5.19)	33.0 (NA)
Median [Min, Max]	45.0 [21.0, 75.0]	56.5 [21.0, 75.0]	44.5 [38.0, 54.0]	33.0 [33.0, 33.0]
**Sex**				
Female	8 (47.1%)	4 (40.0%)	3 (50.0%)	1 (100%)
Male	9 (52.9%)	6 (60.0%)	3 (50.0%)	0 (0%)
**DAS28-CRP (baseline)**				
Mean (SD)	4.90 (1.30)	4.53 (1.34)	5.28 (1.15)	6.32 (NA)
Median [Min, Max]	4.70 [2.50, 7.15]	4.59 [2.50, 7.15]	5.55 [3.77, 6.68]	6.32 [6.32, 6.32]
**DAS28-ESR (baseline)**				
Mean (SD)	5.40 (1.54)	4.89 (1.50)	5.95 (1.39)	7.31 (NA)
Median [Min, Max]	5.21 [2.52, 8.06]	5.08 [2.52, 7.79]	6.12 [4.31, 8.06]	7.31 [7.31, 7.31]
**Treatment**				
DMARD	10 (58.8%)	7 (70.0%)	2 (33.3%)	1 (100%)
Adalimumab	7 (41.2%)	3 (30.0%)	4 (66.7%)	0 (0%)

Supplementary Table 1: Clinical characteristics of patients enrolled in 396.10
